# Targeting and Photodynamic Killing of Cancer Cell by Nitrogen-Doped Titanium Dioxide Coupled with Folic Acid

**DOI:** 10.3390/nano6060113

**Published:** 2016-06-14

**Authors:** Jin Xie, Xiaobo Pan, Mengyan Wang, Longfang Yao, Xinyue Liang, Jiong Ma, Yiyan Fei, Pei-Nan Wang, Lan Mi

**Affiliations:** Department of Optical Science and Engineering, Shanghai Engineering Research Center of Ultra-Precision Optical Manufacturing, Green Photoelectron Platform, Fudan University, 220 Handan Road, Shanghai 200433, China; jinxie13@fudan.edu.cn (J.X.); xiaobopan11@fudan.edu.cn (X.P.); mengyanwang14@fudan.edu.cn (M.W.); yaolongfang@126.com (L.Y.); liangxy14@fudan.edu.cn (X.L.); jiongma@fudan.edu.cn (J.M.); fyy@fudan.edu.cn (Y.F.); pnwang@fudan.edu.cn (P.-N.W.)

**Keywords:** titanium dioxide nanoparticle, photodynamic therapy, cancer cell targeting, folic acid

## Abstract

Titanium dioxide (TiO_2_) has attracted wide attention as a potential photosensitizer (PS) in photodynamic therapy (PDT). However, bare TiO_2_ can only be excited by ultraviolet illumination, and it lacks specific targeting ligands, which largely impede its application. In our study, we produced nitrogen-doped TiO_2_ and linked it with an effective cancer cell targeting agent, folic acid (FA), to obtain N-TiO_2_-FA nanoconjugates. Characterization of N-TiO_2_-FA included Zeta potential, absorption spectra and thermogravimetric analysis. The results showed that N-TiO_2_-FA was successfully produced and it possessed better dispersibility in aqueous solution than unmodified TiO_2_. The N-TiO_2_-FA was incubated with human nasopharyngeal carcinoma (KB) and human pulmonary adenocarcinoma (A549) cells. The KB cells that overexpress folate receptors (FR) on cell membranes were used as FR-positive cancer cells, while A549 cells were used as FR-negative cells. Laser scanning confocal microscopy results showed that KB cells had a higher uptake efficiency of N-TiO_2_-FA, which was about twice that of A549 cells. Finally, N-TiO_2_-FA is of no cytotoxicity, and has a better photokilling effect on KB cells under visible light irradiation. In conclusion, N-TiO_2_-FA can be as high-value as a PS in cancer targeting PDT.

## 1. Introduction

Photodynamic therapy (PDT) based on the photochemical reactions of photosensitizer (PS) is a noninvasive and mild medical treatment for cancer diseases [1,2,3,4]. Titanium dioxide (TiO_2_) nanoparticles (NPs) have been widely used as photocatalysts, photochromics and photovoltaics due to their excellent photoreactivity [5,6,7]. Recently, TiO_2_ has attracted more attention as a potential PS in PDT due to its high chemical stability, excellent oxidation capability and low toxicity [8,9,10]. Under ultraviolet (UV) irradiation, TiO_2_ can be activated and generate cytotoxic reactive oxygen species (ROS). However, UV light is also harmful to normal cells or tissues and has a limited penetration distance in tissues. To enhance the visible light absorption of TiO_2_, various attempts have been made by dye-adsorbed [11,12] or doping methods [13,14]. Moreover, a major challenge for the application of TiO_2_ in PDT is its lack of specific targeting ligands. To improve the cancer cellular uptake of TiO_2_ NPs, specific ligands, such as monoclonal antibodies, peptides and aptamers binding to specific proteins or surface antigens, could be coupled with TiO_2_ NPs. For example, it has been found that folate receptor (FR) is overexpressed on the surfaces of many human tumor cells, whereas it expresses little on the surface of normal cells [15,16]. Folic acid (FA) has high affinity to FR, which can serve as a good tumor marker for targeting treatment in PDT. Although the use of FA to target FR over-expressed tumor cells were studied in depth for various nanomaterials such as ZnO nanoparticles [17] and Au nanorods [18], FA functionalized TiO_2_ NPs with enhanced visible light absorption has not been explored. Recently, the Lee Wen-Chien team modified FA on TiO_2_ NPs, and used the obtained FA/TiO_2_ as a PS under UV light [19]. However, UV light itself can have a photokilling on all cells, so it is not encouraged in PDT application. Herein, we reported a promising PS, nitrogen-doped TiO_2_ with FA on its surface (N-TiO_2_-FA). The prepared N-TiO_2_-FA NPs were characterized by Zeta potential, absorption spectra and thermogravimetric analysis. In this study, human nasopharyngeal carcinoma (KB) cells and human pulmonary adenocarcinoma (A549) cells were used as FR-positive and FR-negative cell lines, respectively. The targeting efficiency of N-TiO_2_-FA for different cells was studied by laser scanning confocal microscopy. Finally, the cytotoxicity and photokilling effect of N-TiO_2_-FA under visible light irradiation were researched.

## 2. Results

### 2.1. Preparation and Characterization of N-TiO_2_-FA

**Figure 1:**
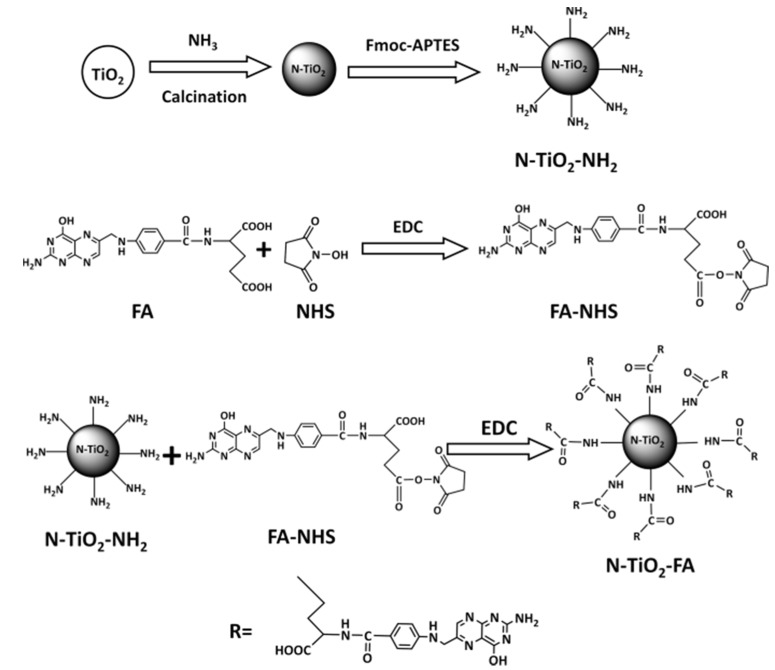
Synthesis procedure of N-TiO_2_-folic acid (FA) nanoparticles (NPs).

Figure 1 shows the synthesis procedure of N-TiO_2_-FAs. The nitrogen-doping TiO_2_ (N-TiO_2_) NPs were first obtained by calcination of pure TiO_2_ NPs in ammonia atmosphere for higher visible light absorbance in the range of 400–550 nm [20]. Using amino silanization method [21], the N-TiO_2_ NPs were modified to obtain positively charged N-TiO_2_-NH_2_ NPs, whose surfaces were full of the protonated amino groups (-NH_3_^+^). FA and N-Hydroxysuccinimide (NHS) were reacted to form NHS-FA, then FA was conjugated with N-TiO_2_-NH_2_ following a standard procedure [22]. It should be noted that FA has α- and γ-carboxylic acids and both of them could react with NHS. In such a reaction, the γ-carboxylic acid is primarily conjugated due to its higher reactivity [23].

**Figure 2:**
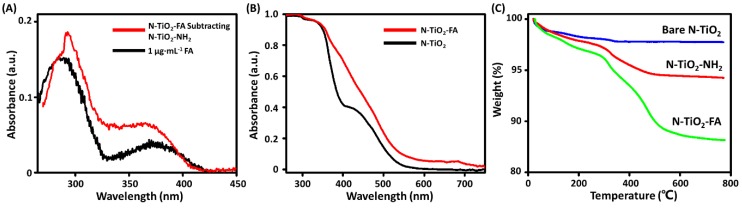
(**A**) Comparison of the absorption spectrum of 1 μg·mL^−1^ folic acid (FA) solution and the subtraction spectrum of N-TiO_2_-FA subtracting N-TiO_2_-NH_2_; (**B**) Absorption spectra of N-TiO_2_ and N-TiO_2_-FA NPs; (**C**) Thermogravimetric analysis of the air-dried N-TiO_2_, N-TiO_2_-NH_2_ and N-TiO_2_-FA NPs.

The absorbance spectra of N-TiO_2_-FA and N-TiO_2_-NH_2_ solutions were measured and analyzed. The absorption spectrum of N-TiO_2_-FA subtracting that of N-TiO_2_-NH_2_ with the same concentration of 10 μg·mL^−1^ was shown in Figure 2A. It can be seen that the subtracting spectrum was very similar with the spectrum of 1 μg·mL^−1^ FA solution, which indicated that FA was successfully linked with the NPs. As shown in Figure 2B, the absorption spectra of N-TiO_2_ and N-TiO_2_-FA NPs were both in the visible region between 400 and 550 nm. The absorption of N-TiO_2_ was similar to the previous report due to nitrogen-doping [20]. N-TiO_2_-FA NPs demonstrated enhanced absorbance respect to N-TiO_2_, which may be attributed to the surface modifier. The higher absorption of N-TiO_2_-FA might lead to higher photoactivity under visible light. The thermogravimetric analysis (TGA) of the air-dried N-TiO_2_, N-TiO_2_-NH_2_, and N-TiO_2_-FA NPs were shown in Figure 2C. The weight losses at 800 °C of N-TiO_2_, N-TiO_2_-NH_2_, and N-TiO_2_-FA NPs were 2.26, 5.75, and 11.87 wt %, respectively. The weight loss of N-TiO_2_-NH_2_ NPs with respect to N-TiO_2_ was 3.49 wt %, which was due to the amino silane coupling agent. It could be estimated that the conjugated amount of -NH_2_ on one N-TiO_2_ NP was 205 by assuming N-TiO_2_ NPs were spheres with the diameter of 10 nm. The weight loss of N-TiO_2_-FA NPs with respect to N-TiO_2_-NH_2_ was 6.12 wt %, which was due to the disappearance of FA. Thus, the conjugated amount of FA on one N-TiO_2_ NP was further estimated about 193. According to the TGA result, one can conclude that almost all of the amino coupling agents were conjugated with FA. 

The surface electrical property of samples was studied by Zeta potential measurement. It is well known that when the absolute value of Zeta potential is above 30 mV, the NPs can be stably dispersed in the solvent [24]. The Zeta potential of N-TiO_2_ was −10.4 ± 0.6 mV, which was negatively charged and with poor dispersibility. Capping with cationic amino groups, the Zeta potential of the positively charged N-TiO_2_-NH_2_ was +20.6 ± 2.3 mV. As FA conjugated on N-TiO_2_ NPs, N-TiO_2_-FA became negatively charged (−27.4 ± 1.2 mV) and more stably dispersed in solution at pH = 7. These results confirmed that FA was successfully conjugated with N-TiO_2_ NPs and implied that the obtained N-TiO_2_-FA NPs had good dispersibility.

### 2.2. Cellular Uptake

**Figure 3:**
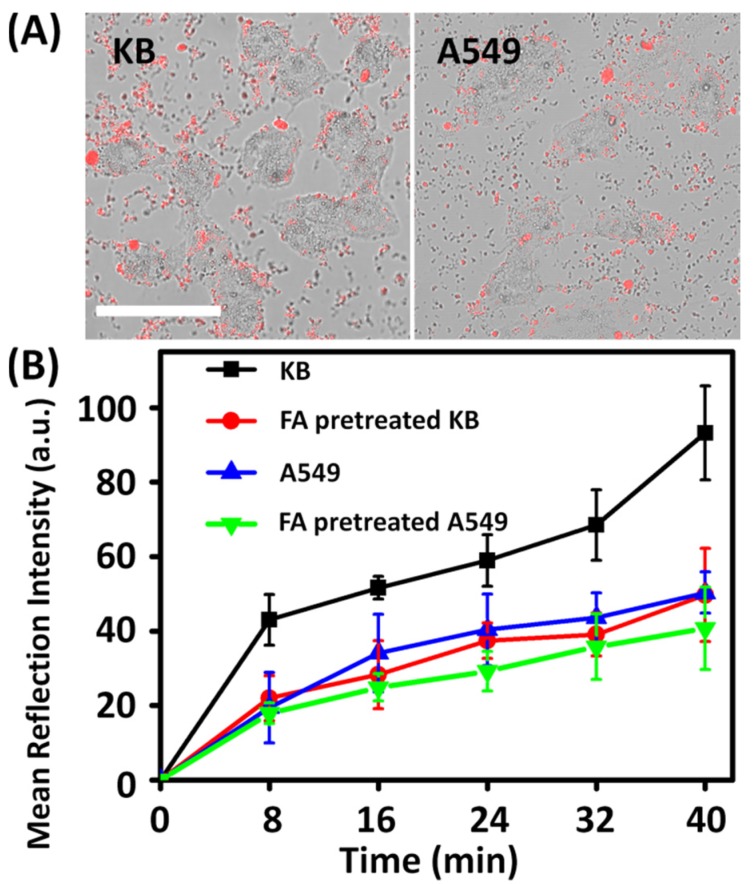
(**A**) Confocal microscopic images of human nasopharyngeal carcinoma (KB) and human pulmonary adenocarcinoma (A549) cells treated with N-TiO_2_-FA (red) for 40 min. Scale bar is 50 μm. (**B**) Reflection intensity of internalized N-TiO_2_-FA in KB (black), FA-pretreated KB (red), A549 (blue) cells and FA-pretreated A549 (green) as a function of incubation time.

The cellular uptake of unmodified TiO_2_ NPs is inefficient due to their poor dispersibility, while N-TiO_2_-FA NPs with improved dispersibility can enter cells. According to our previous result, TiO_2_ NPs could enter cells non-specifically [24]. It was reported that the negatively charged TiO_2_ NPs entered HeLa cells by various pathways such as caveolin-mediated endocytosis, clathrin-mediated endocytosis, and micropinocytosis. When cells were incubated in 4 °C, there were still some TiO_2_ NPs entering HeLa cells [24]. In this study, KB cells as the FR-positive cancer cells and A549 cells as the FR-negative cancer cells were cultured to investigate the cellular uptake of N-TiO_2_-FA with the same condition. After 40 min incubation with culture medium containing 50 μg·mL^−1^ N-TiO_2_-FA, KB cells exhibited higher reflection intensity of internalized N-TiO_2_-FA than A549 cells as shown in Figure 3A. Moreover, it can be seen that some of N-TiO_2_-FA NPs were around the cell membranes of A549, instead of inside. This suggests that the cellular uptake of TiO_2_-FA NPs was more effective for FR-positive KB cells. The average amount of N-TiO_2_-FA NPs located in KB, A549 and FA-pretreated KB or A549 cells with increasing incubation time were quantified by analyzing the reflection intensity of N-TiO_2_-FA NPs inside individual cells. As shown in Figure 3B, the amounts of endocytosed N-TiO_2_-FA NPs in KB cells were about twice that in A549 cells and FA-pretreated cells. For the FA-pretreated KB cells, the receptors of KB cells were occupied by loaded FA, so the amount of N-TiO_2_-FA in FA-pretreated KB cells was similar with those of A549 cells. This result also confirmed that FA was successfully linked with N-TiO_2_ NPs and the N-TiO_2_-FA NPs could target FR-positive cancer cells with high efficiency. Figure 3B showed that the amount of N-TiO_2_-FA in FA-pretreated A549 cells decreased by around 10% compared with non-pretreated A549 cells. Therefore, it can be concluded that FA can only block a small part of uptake of N-TiO_2_-FA NPs for A549 cells. We also incubated KB and A549 cells with N-TiO_2_ for 40 min and found that the amounts of N-TiO_2_ in cells were similar to those of FA-pretreated ones. This result proved that the coupled FA on N-TiO_2_ NPs improved the uptake efficiency of NPs for FR-positive cancer cells. 

### 2.3. Cytotoxicity of N-TiO_2_-FA

**Figure 4:**
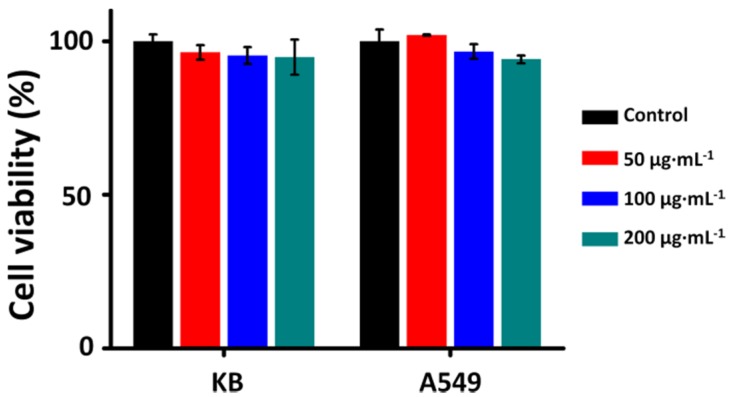
The dark cytotoxicity of N-TiO_2_-FA with the incubation concentrations of 50–200 μg·mL^−1^ on KB and A549 cells. The control groups of untreated cells were also shown for comparison. Data are expressed as mean ± SD (*n* = 4).

The cell viability assays of KB and A549 cells were measured after the cells were incubated with 50–200 μg·mL^−1^ N-TiO_2_-FA for 1 h in order to evaluate its cytotoxicity. As shown in Figure 4, all the surviving fractions of the treated cells were greater than 90%. It can be concluded that nitrogen-doping, surface-modification or coupling with FA has little influence on cytotoxicity. These results also showed that the cytotoxicity of the N-TiO_2_-FA NPs is quite low and negligible, indicating that N-TiO_2_-FA NPs are well biocompatible, safe in dark, and of therapy application value.

### 2.4. Targeting and Photokilling Effect on Cancer Cells

**Figure 5:**
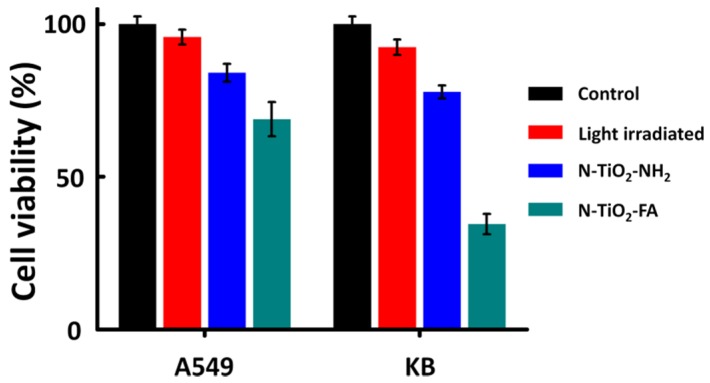
Photokilling effects of N-TiO_2_-FA (green) and N-TiO_2_-NH_2_ (blue) with the concentration of 200 μg·mL^−1^ on A549 and KB cells. The control group (black) and light irradiated group (red) were also shown for comparison. Data are expressed as mean ± SD (*n* = 4).

The photokilling effects were measured under visible-light irradiation (400–575 nm) of the light dose 190 J·cm^−2^. The surviving fractions of KB and A549 cells in dependence on the kinds of photosensitizer samples were shown in Figure 5. N-TiO_2_-NH_2_ showed a weak and similar photokilling effect on both cell lines with about 80% survival fractions. N-TiO_2_-FA showed a higher photokilling effect on A549 cells with 68.9% cell viability which is due to its better dispersibility with the comparison of N-TiO_2_-NH_2_. However, N-TiO_2_-FA exhibited an excellent photokilling effect on KB cells with the cell viability dropping to 34.5%. Compared with N-TiO_2_-NH_2_, the N-TiO_2_-FA NPs demonstrated the targeting specificity for FR and exhibited a better photokilling effect on FR-positive tumor cells, which we can attribute to the coupling with FA.

## 3. Discussion

Wang *et al.* [25] evaluated the effectiveness of nano-TiO_2_-based PDT with UV light on glioma. They conducted the experiment on female BALB/c-nude mice with the treatment solutions containing 200 μg·mL^−1^ TiO_2_ and the dosage of TiO_2_ in mice was about 10 mg·kg^−1^. You *et al.* [26] conducted sonodynamic therapy using TiO_2_ with the dosage of 100 μg·mL^−1^ *in vitro* and 5 mg·kg^−1^ in mice. Considering these reports, the solution concentration of 200 μg·mL^−1^ in this study could be therapeutic *in vivo* and the dosage of NPs should be decided according to the weight of the experiment animals.

Compared with the much more common gold nanorods [27], the cetyltrimethylammonium bromide (CTAB) as the initial stabilizer of gold nanorods exhibits inevitable toxicity in biological issues [28]. Additionally, the nitrogen-doped TiO_2_ particles can be prepared fruitfully by an easily operative method with much lower cost. Moreover, the nitrogen-doped TiO_2_ particles possess the photosensitizing properties in visible light, while gold nanorods were often used as the carrier of the tradition photosensitizers [29,30]. Therefore, nitrogen-doped TiO_2_ nanoparticles were of high value in therapy applications.

## 4. Materials and Methods

### 4.1. Materials

Anatase TiO_2_ NPs (Sigma-Aldrich Inc., St. Louis, MO, USA) with a nominal diameter less than 15 nm were used in this study. An activated silane coupling agent 3-aminopropyltriethoxysilane (APTES, 99%, Aladdin Inc., Shanghai, China) was used for positive charge modification of TiO_2_ NPs. Other chemical agents involved in positive charge modification include Dimethylformamide (DMF, 98%, Sinopharm Chemical Reagent Co., Ltd., Shanghai, China), 2-(9H-fluoren-9-ylmethoxycarbonylamino)oxyacetic acid (Fmoc-Aoa, Chem-Impex International, Inc., Wood Dale, IL, USA), *N*,*N*-Diisopropylethylamine (DIPEA, 99.5%, Sigma-Aldrich Inc., St. Louis, MO, USA), (Benzotriazole-1-yloxy)tripyrrolidinophosphonium hexafluorophosphate (PyBOP, 98%, EMD Chemicals, Inc., Gibbstown, NJ, USA), piperidine (≥99.5%, Sigma-Aldrich Inc., St. Louis, MO, USA), and ammonium solution (25%–28%, Tongsheng Inc., Yixing, China). The chemical agents used in the conjugation of folic acid on TiO_2_ NPs were folic acid (FA, ≥97%, Sigma-Aldrich), dimethyl sulfoxide (DMSO, ≥99.7%, Sigma-Aldrich), 1-(3-Dimethylaminopropyl)-3-ethylcarbodiimide hydrochloride (EDC, 99%, Sigma-Aldrich), and *N*-Hydroxysuccinimide (NHS, 99%, Sigma-Aldrich).

### 4.2. Preparation of N-TiO_2_-FA

The anatase TiO_2_ NPs were first calcinated in ammonia atmosphere to obtain nitrogen-doping TiO_2_ (N-TiO_2_) NPs as described in our previous work [20]. The N-TiO_2_ nanoparticles were modified with APTES to obtain positively charged N-TiO_2_-NH_2_ NPs following the procedure in our previous work [24]. In brief, 0.7 mL APTES, 0.94 mg Fmoc-Aoa (1 mM), 3.122 mg PyBOP (2 mM) and 1 μL DIPEA (0.5 mM) were dispersed in 2.3 mL DMF, followed by stirring for 12 h at room temperature. Then 0.7 mg TiO_2_, 0.7 mL ammonia water and 3.3 mL DMF were added into the stirred solution. The produced suspension was sonicated for 20 min and stirred for another 24 h before adding 70 μL redistilled piperidine. The positively charged N-TiO_2_-NH_2_ was obtained by stirring the reaction solution for 12 h. Subsequently, FA (0.011 mM), EDC (0.003 mM) and NHS (0.003 mM) with a mole ratio of 10:3:3 were placed in a flask and dissolved in 20 mL DMSO to prepare NHS-FA, and then 1 mg N-TiO_2_-NH_2_ was dispersed in it, followed by an ultrasonic dispersion for 15 min. The mixture solution was stirred to react at room temperature for 12 h. The N-TiO_2_-FA sample was separated from the reaction mixture by centrifugation at 8000 rpm for 30 min and washed by DMSO once and by deionized water twice to remove the excess FA, EDC and NHS. 

### 4.3. Characterization of Samples

The Zeta potentials of N-TiO_2_-FA, N-TiO_2_-NH_2_, as well as bare TiO_2_ NPs dissolved in deionized water with the concentration of 50 μg·mL^−1^ were measured by Zetasizer Nano ZS90 (Malvern Instruments; Worcestershire; UK) at 25 °C and pH = 7. Absorption spectra of the samples were measured using a spectrometer (Shimadzu, UV3101pc; Japan). Thermogravimetric analysis (TGA) for the air-dried N-TiO_2_, N-TiO_2_-NH_2_ and N-TiO_2_-FA NPs was performed on a SDT Q600 (TA Instrument, New Castle, DE, USA) from 20 to 800 °C at a heating rate of 10 °C·min^−1^ under N_2_ flow.

### 4.4. Cell Culture

KB and A549 cells were cultured in RPMI-1640 medium (Roswell Park Memorial Institute 1640, Gibco, New York, NY, USA) with 10% (*v*/*v*) fetal bovine serum (Sijiqing Inc., Zhejiang, China) in a humidified standard incubator with a CO_2_ atmosphere at 37 °C. The cells were seeded in Petri dishes or 96-well plates according to experimental needs. All experiments were performed after the cells have reached 80% confluence with normal morphology. 

### 4.5. Cellular Uptake Study of N-TiO_2_-FA

KB or A549 cells in Petri dishes were incubated with RPMI-1640 medium containing 50 μg·mL^−1^ TiO_2_-FA for 8, 16, 24, 32, or 40 min. Then, the medium containing surplus TiO_2_ NPs was removed and changed to fresh culture medium. For comparison, a dish of KB cells and a dish of A549 cells were treated with 500 μg·mL^−1^ FA in RPMI-1640 medium for 2 h before being treated with N-TiO_2_-FA NPs by the same method.

Cells in each experiment were fixed with 4% paraformaldehyde in phosphate buffered saline (PBS) solution for 10 min and were washed three times using PBS before observation by a laser scanning confocal microscope (LSCM, Olympus, FV300/IX 71, Tokyo, Japan), which is equipped with a 488 nm Ar^+^ continuous laser excitation (Coherent, Santa Clara, CA, USA) and a water-dipping objective (60×, NA = 1.2).

The cellular uptake of N-TiO_2_-FA and N-TiO_2_ NPs were observed by the LSCM. The reflection images of the TiO_2_ NPs were obtained through a channel of the microscope without filter. The differential interference contrast (DIC) micrographs were observed simultaneously in a transmission channel to demonstrate the cell morphology and identify the borders of the cells. The quantitative study of N-TiO_2_-FA NPs was analyzed using an open source software ImageJ to evaluate the reflection intensity of NPs inside individual cells. At least 50 cells were analyzed for each condition.

### 4.6. Measurements of Photokilling Effect and Cytotoxicity

KB or A549 cells grown in 96-well plates (1 × 10^5^ cells per well) were incubated with TiO_2_-FA NPs dispersed in RPMI-1640 medium with different concentrations between 50 and 200 μg·mL^−1^ for 1 h in the dark. Then the medium was removed and replaced by fresh culture medium. 

To study the photokilling effect, the cells were irradiated by an 18-W tungsten halogen lamp. Two pieces of quartz lens were used to obtain a concentrated parallel light beam and two filters were used to obtain a light beam of 400–575 nm. The visible-light illumination dose for cells was 190 J·cm^−2^. After irradiation, the cells were incubated in the dark for 24 h before further study. The cytotoxicity examinations were carried out with the same procedure as the photokilling effect but without irradiation.

The cell viability assays were conducted by a modified MTT method using WST-8 (2-(2-methoxy-4-nitrophenyl)-3-(4-nitrophenyl)-5-(2, 4-disulfophenyl)-2H tetrazolium, monosodium salt, Beyotime, Jiangsu, China). Each well containing 100 μL culture medium was added with 10 μL of WST-8. The cells were incubated at 37 °C with 5% CO_2_ for 2 h and the absorbance was measured at 450 nm using a microplate reader (Bio-Tek Instruments Inc., Winooski, VT, USA). To diminish the influence of the NPs, the absorbance values at 450 nm before adding WST-8 dyes were also measured as a reference. Cells incubated in RPMI-1640 medium without any treatment were used as the control group. Each experiment was conducted and measured independently for at least three times.

## 5. Conclusions

In this study, we have successfully produced N-TiO_2_-FA nanoconjugates. Zeta potential results showed that N-TiO_2_-FA had a negative Zeta potential and possessed good dispersibility. The absorption spectra and TGA result showed that FA was connected to the surface of N-TiO_2_ NPs. The incubation experiment results showed that N-TiO_2_-FA had higher uptake efficiency in FR-positive KB cells than FR-negative A549 cells, as well as the FA-pretreated cells. Finally, N-TiO_2_-FA had no obvious cytotoxicity, but had a significant photokilling effect on FR-positive KB cells with visible light illumination. Therefore, N-TiO_2_-FA can be of high value as a PS for cancer targeting PDT.
